# Phenomenology of future-oriented mind-wandering episodes

**DOI:** 10.3389/fpsyg.2013.00425

**Published:** 2013-07-16

**Authors:** David Stawarczyk, Helena Cassol, Arnaud D'Argembeau

**Affiliations:** ^1^Department of Psychology - Cognition and Behavior, University of LiègeLiège, Belgium; ^2^Fund for Scientific Research (FRS-FNRS)Brussels, Belgium

**Keywords:** mind-wandering, phenomenology, planning, future thinking, factorial structure

## Abstract

Recent research suggests that prospective and non-prospective forms of mind-wandering possess distinct properties, yet little is known about what exactly differentiates between future-oriented and non-future-oriented mind-wandering episodes. In the present study, we used multilevel exploratory factor analyses (MEFA) to examine the factorial structure of various phenomenological dimensions of mind-wandering, and we then investigated whether future-oriented mind-wandering episodes differ from other classes of mind-wandering along the identified factors. We found that the phenomenological dimensions of mind-wandering are structured in four factors: representational format (inner speech vs. visual imagery), personal relevance, realism/concreteness, and structuration. Prospective mind-wandering differed from non-prospective mind-wandering along each of these factors. Specifically, future-oriented mind-wandering episodes involved inner speech to a greater extent, were more personally relevant, more realistic/concrete, and more often part of structured sequences of thoughts. These results show that future-oriented mind-wandering possesses a unique phenomenological signature and provide new insights into how this particular form of mind-wandering may adaptively contribute to autobiographical planning.

## Introduction

Mind-wandering occurs when attention drifts away from the task at hand and focuses on internally-generated thoughts that are not directly related to the present environment, such as memories or prospective thoughts (Singer, 1993; Smallwood and Schooler, 2006; Schooler et al., 2011; Stawarczyk et al., 2011a,b). This phenomenon is quite frequent, representing from 20 to 50 percent of our daily thinking time (Kane et al., 2007; Killingsworth and Gilbert, 2010; Song and Wang, 2012), and research has shown that it generally impairs current task performance (Smallwood et al., 2007; McVay and Kane, 2010; Smallwood, 2011) and is associated with decreased processing of stimuli from the external environment (Barron et al., 2011; Braboszcz and Delorme, 2011; Kam et al., 2011). These findings suggest that mind-wandering represents a state of decoupled attention from the external world during which attentional resources are directed toward the processing and maintenance of internally generated thoughts (Smallwood and Schooler, 2006; Smallwood, 2010, 2013; Schooler et al., 2011; Smallwood et al., 2012).

Although mind-wandering has recently received increased interest in cognitive psychology and neuroscience (for recent reviews, see Klinger, 2009; Christoff et al., 2011; Smallwood, 2011; Andrews-Hanna, 2012; Christoff, 2012; Fell, 2012; Kane and McVay, 2012; Mooneyham and Schooler, 2013), there is surprisingly little data on the content and phenomenological features of mind-wandering episodes. A few studies have explored some phenomenological characteristics of mind-wandering but, for most of them, these research exclusively focused on a single dimension, such as the representational format of thoughts (Antrobus et al., 1966, 1970), their affective valence (Marchetti et al., 2012), the spontaneous nature of their occurrence (Giambra, 1995; Forster and Lavie, 2009), or their structuration in complex sequences (Teasdale et al., 1993; Stuyven and Van Der Goten, 1995). Other studies have shown that most mind-wandering episodes are oriented toward the future (Smallwood et al., 2009; Stawarczyk et al., 2011a; Song and Wang, 2012), and that these future-oriented mind-wandering episodes are mostly self-related and goal-directed—they mainly involve autobiographical planning (Stawarczyk et al., 2011a; Song and Wang, 2012)—whereas the goal-directedness of past-oriented mind-wandering is much less marked (Baird et al., 2011). The evidence further suggests that prospective mind-wandering might possess specific properties. For instance, it has been shown that the frequency of future-oriented mind-wandering is specifically increased by previous reflections upon one's personal goals (Stawarczyk et al., 2011a) and self-related traits (Smallwood et al., 2011), whereas the frequency of past-oriented mind-wandering remains unchanged. In addition, some studies have shown that prospective mind-wandering makes more demands on processing resources than non-future-oriented mind-wandering: with increasing task demands, future-oriented mind-wandering decreases to a larger extent than past-oriented mind-wandering (Smallwood et al., 2009), and individuals with higher working memory capacity report more future-oriented mind-wandering episodes but not more past- or present-oriented episodes during relatively undemanding tasks (Baird et al., 2011).

Current evidence thus suggests that different types of mind-wandering episodes possess distinct properties and, in particular, that prospective mind-wandering differs from other forms of mind-wandering. Little is known, however, about what exactly differentiates prospective from non-prospective forms of mind-wandering. Studies of “directed” future thinking (i.e., studies in which the participants are explicitly instructed to form mental representations of the future) have shown that prospective thoughts differ from past thoughts along several phenomenological features, including levels of sensory details (D'Argembeau and Van Der Linden, 2004, 2006), emotional valence (Macleod and Byrne, 1996; D'Argembeau and Van Der Linden, 2006; Rasmussen and Berntsen, 2013), and personal relevance (D'Argembeau and Van Der Linden, 2006; Addis et al., 2008; Berntsen and Bohn, 2010). Whether similar phenomenological differences characterize prospective and non-prospective forms of mind-wandering is currently unknown. The phenomenological qualities of mental representations play important roles in determining one's conviction (accurate or not) that they are tied to reality (Johnson, 1988), which in turn may influence decision making and behavior (Johnson and Sherman, 1990; Roese and Sherman, 2007). From this perspective, a better understanding of the phenomenological structure of mind-wandering, and of how various forms of mind-wandering differ along important phenomenological dimensions, may provide important insight into the beneficial and deleterious effects of mind-wandering episodes (for a recent review on the costs and benefits of mind-wandering, see Mooneyham and Schooler, 2013).

To seek insight into these questions, the current study sampled the occurrence and content of mind-wandering episodes with thought-probes during the Sustained Attention to Response Task (SART; Robertson et al., 1997). After the SART, participants were asked to rate each reported mind-wandering episode on various phenomenological dimensions that could potentially differentiate a range of mind-wandering episodes, including their representational format (Antrobus et al., 1970; Klinger and Cox, 1987; Heavey and Hurlburt, 2008; Delamillieure et al., 2010; Song and Wang, 2012), structuration and intentional aspect (Teasdale et al., 1993; Giambra, 1995; Stuyven and Van Der Goten, 1995; Forster and Lavie, 2009), repetitiveness, abstractness, and emotional valence (Watkins, 2008, 2010), and links with personal goals and concerns (Klinger, 1978, 1999, 2009; Klinger et al., 1980; Gold and Reilly, 1985). These phenomenological ratings were submitted to an exploratory multilevel factor analysis in order to determine the factorial structure of mind-wandering characteristics both at the within-participant and between-participant levels (Muthén, 1994; Reise et al., 2005). We then compared future-oriented mind-wandering episodes with non-future-oriented mind-wandering episodes along the dimensional factors revealed by the multilevel factor analysis. Additionally, we explored whether the features of future-oriented mind-wandering differ according to the temporal distance of represented events.

## Methods

### Participants

A total of 67 participants (32 men) from the Belgian general population volunteered to participate in the study. Their age ranged from 18 to 30 years with a mean age of 23.28 years (*SD* = 2.08). Individuals with medical, neurological, or psychiatric disorders were excluded from the study. This study was part of a broader research project that was approved by the Ethical Committee of the faculty of Psychology and Education of the University of Liège. All participants provided written informed consent.

### Task and questionnaires

#### SART with thought-probes

Participants completed a version of the SART with thought-probes adapted from Stawarczyk et al. (2011a). Stimuli (numbers between 1 and 9) were presented sequentially at the center of the screen. Participants were asked to respond as fast and accurately as possible to the numbers and to withhold their response when presented with the number 3 (the target stimulus). The probability of the target stimulus was 11%. The interstimulus interval was 2000 ms, and the duration of each stimulus was 500 ms. The task comprised 30 blocks whose duration was either 25, 35, 45, 55, or 65 s. Six blocks of each length were presented in a predetermined pseudorandom order. Across the 30 blocks, 540 numbers (both targets and non-targets) were presented for a total duration of 22 min and 30 s. Each block was immediately followed by a thought-probe which interrupted the task. For each of these interruptions, a question appeared on the screen asking participants whether they had been experiencing mind-wandering episodes since the last interruption (or since the beginning of the task for the first interruption). Participants were explained in details that mind-wandering episodes specifically referred to stimulus-independent and task-unrelated thoughts and thus other experiences were excluded from this category, including thoughts related to the appraisal of the task (i.e., task-related interferences) and distractions by currently experienced exteroceptive perceptions and interoceptive sensations (i.e., external distractions; see Stawarczyk et al., 2011a). After responding to each probe with a key press (“o” for yes or “n” for no), a short text was displayed on the screen which asked participants to press the response key (the spacebar) to continue the task. When the participants gave a positive answer to the thought-probes, the text display furthermore reminded them to write a brief description of all the mind-wandering episodes they had experienced since the last interruption. Participants were told that their descriptions should be detailed enough to allow them to clearly remember after the task what they had thought about during each mind-wandering episode. No mention was made about the Thought Characteristics Questionnaire at this stage. Before beginning the SART, participants performed a short training session of the task and were given a written summary of the instructions to help them in case of doubt when responding to the thought-probes.

#### Thought characteristics questionnaire (TCQ)

The content and characteristics of each mind-wandering episode reported during the SART were assessed with a self-report questionnaire adapted from the Memory Characteristics Questionnaire created by Johnson et al. (1988) and previously used in Stawarczyk et al. (2011a). For each mind-wandering episode, the following phenomenological dimensions were assessed with seven-point Likert scales: (1) the thought involved visual imagery (1 = *not at all*, 7 = *totally*), (2) the thought involved inner speech (1 = *not at all*, 7 = *totally*), (3) the occurrence of the thought was intended and intentional (i.e., the participant intentionally decided to think of something else than the SART; 1 = *not at all*, 7 = *totally*), (4) the thought belonged to a structured succession of thoughts (such as in reasoning, reflection or argumentation; 1 = *not at all*, 7 = *totally*), (5) the content of the thought was realistic and plausible (1 = *not at all*, 7 = *totally*), (6) the content of the thought was related to something concrete and well-defined (e.g., a precise situation or a particular action; 1 = *not at all*, 7 = *totally*), (7) the content of the thought was of importance to the participant's life (1 = *not at all*, 7 = *totally*), (8) the content of the thought was related to the participant's personal goals (1 = *not at all*, 7 = *totally*), (9) the thought often comes to the participant's mind in daily life (1 = *never*, 7 = *very often*), and (10) the affective valence of the thought's content (−3 = *very negative*, +3 = *very positive*).

In addition to these phenomenological dimensions, participants were also asked to characterize each mind-wandering episode according to its temporal orientation by choosing between: (1) past, (2) present, (3) future or (4) no precise temporal orientation. For past and future mind-wandering episodes, participants were also asked to specify the temporal distance of their thoughts by choosing between six different categories: (1) before/later in the present day, (2) between yesterday/tomorrow and the past/next 7 days, (3) between 1 week and 1 month in the past/future, (4) between 1 month and 1 year in the past/future, (5) more than 1 year away in the past/future and (6) no precise temporal distance. Finally, subjects were asked to specify the main function of each thought by choosing between: (1) to make a decision/solve a problem, (2) to plan something, (3) to reappraise a situation, (4) to make the participant feel better, (5) to keep the participant aroused, (6) another non-listed function (in which case, participants were asked to specify what the function was), and (7) daydream with no apparent function.

## Results

### SART performances and thought-probe responses

The mean proportion of correct responses to the target stimuli was 66.57% (*SD* = 18.16), the mean reaction time (RT) to the non-targets was 348 ms (*SD* = 41), and the mean coefficient of variation (CV; the ratio of the standard deviation to the mean) of RTs for the non-targets was 20.63 (*SD* = 5.23). Participants reported having experienced mind-wandering episodes for a mean of 30.60% of the thought-probes (*SD* = 19.11; range = 3.33–86.67%), with a mean of 11.04 reported thoughts per participants (*SD* = 7.94; range = 1–39). In total, 740 mind-wandering episodes were reported across participants and had their content and characteristics evaluated on the TCQ.

Analyses of SART performance according to the responses given to the thought probes revealed that response accuracy to the targets was lower [*t*_(66)_ = 2.51; *p* = 0.01; no MW = 68.01% ± 19.45; MW = 61.52% ± 23.75], RT for the non-targets was slower [*t*_(66)_ = −3.56; *p* < 0.001; no MW = 346 ms ± 40; MW = 353 ms ± 45], and CV for the non-target was larger [*t*_(66)_ = −3.12; *p* < 0.01; no MW = 20.10 ± 5.15; MW = 21.67 ± 6.22] when the participants reported having experienced mind-wandering. Across the entire task, there was a negative correlation between reported mind-wandering episodes and target accuracy (*r* = −0.30; *p* = 0.01), as well as a positive correlation between mind-wandering and CV of RTs for non-targets (*r* = 0.37; *p* = 0.002). The correlation between mind-wandering episodes and mean RTs was not significant (*r* = 0.09; *p* = 0.45). Together, these findings replicate those of previous studies (McVay and Kane, 2009; Stawarczyk et al., 2011a; McVay and Kane, 2012) and demonstrate the validity of the subjective reports of mind-wandering made by the participants in the present research.

### Phenomenology of mind-wandering episodes

#### Descriptive statistics of the TCQ dimensions

**Figure 1:**
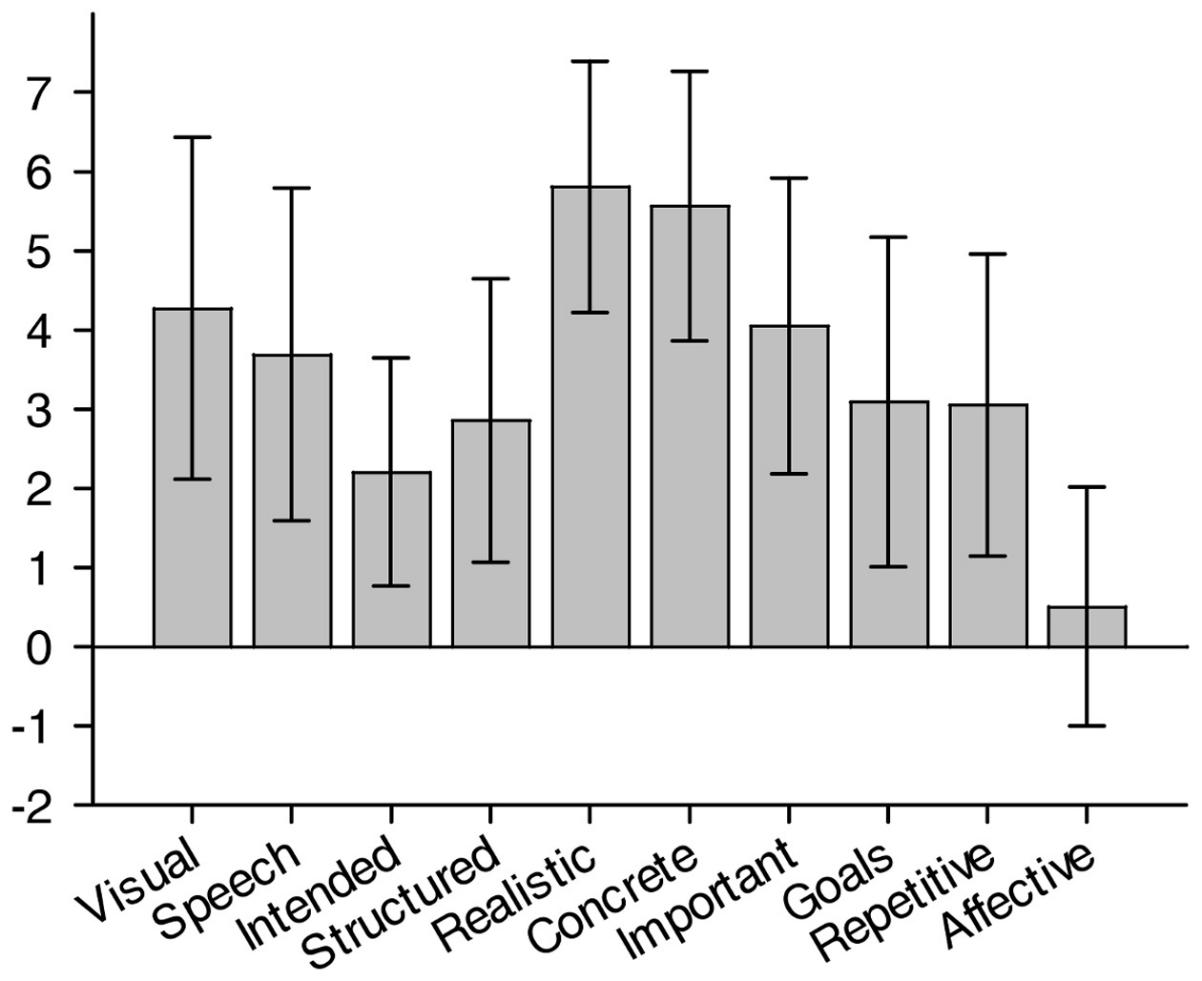
**Descriptive statistics of the TCQ dimensions**. Note: all dimensions were rated on scales ranging from 1 to 7 except the affective valence scale which ranged from −3 to +3. Bars represent the standard deviation of the mean.

We first present descriptive statistics for the various phenomenological dimensions of the TCQ. The mean scores and standard deviation for each TCQ dimension calculated for the 740 mind-wandering episodes across all participants are presented in Figure 1. These descriptive statistics show that, on average, mind-wandering episodes involved a moderate amount of visual imagery and inner speech, and that, for the most part, they did not belong to a structured sequence of thoughts and their occurrence was not intended. Furthermore, mind-wandering content was mostly realistic and concrete, moderately important, and not necessarily strongly related to the participants' personal goals (although there was substantial variability in this respect). Finally, most of the reported mind-wandering episodes did not involve thoughts that occur repetitively, and their affective content was neutral, although showing a slight positive bias. Together, these results largely replicate those of a previous study that also used the TCQ to assess the phenomenological dimensions of mind-wandering (Stawarczyk et al., 2011a).

#### Multilevel factor analyses of the TCQ dimensions

Next, we sought to investigate the internal structure of mind-wandering phenomenology, that is, the functional dependencies and independence among the various dimensions investigated in this study. Specifically, we examined whether some of the phenomenological dimensions of mind-wandering are related to each other at the intra-individual level (i.e., whether the scores on some dimensions tend to vary together within participants) and at the inter-individual level (i.e., whether participants with higher scores on one dimension tend to have higher scores on some other dimensions). To investigate these two issues, the data from the 740 mind-wandering episodes (which are nested within the 67 participants) were submitted to a multilevel exploratory factor analysis (MEFA; Muthén, 1994; Reise et al., 2005) computed with Mplus 6.11 (Muthén and Muthén, 1998-2010). MEFA separately provides the factor structure of the data at the within- and between-individual levels, and is performed in a succession of steps that are described below.

The first step is to examine the intraclass correlation (ICC) for each measured item in order to determine whether the MEFA is necessary. The ICC estimates the amount of the total item variance that is due to between-individual variance. ICC varies between zero and one; an ICC of zero indicates that all the variation is within individuals, whereas an ICC of one indicates that all the variation is between individuals. It has been suggested that MEFA are required to properly examine the factor structure of nested data when the ICC of the items is above 0.05 (Reise et al., 2005). The ICC results for the present data are provided in Table 1 and show that approximately between 20% and 40% of the item variance is between individuals, confirming the necessity of MEFA to properly analyze the data in the present study.

**Table 1:** **Within and between correlation matrices, and intraclass correlations for TCQ items**.

	**1**	**2**	**3**	**4**	**5**	**6**	**7**	**8**	**9**	**10**
**MAXIMUM LIKELIHOOD ESTIMATED SIGMA WITHIN CORRELATION MATRIX**										
1. Visual	1									
2. Speech	−0.55	1								
3. Intended	−0.08	0.19	1							
4. Structured	−0.19	0.33	0.19	1						
5. Realistic	−0.05	0.10	0.04	0.07	1					
6. Concrete	−0.03	0.12	0.08	0.10	0.52	1				
7. Important	−0.14	0.26	0.08	0.19	0.27	0.14	1			
8. Goals	−0.24	0.34	0.08	0.23	0.22	0.14	0.61	1		
9. Repetitive	−0.15	0.25	0.05	0.12	0.12	0.01	0.57	0.52	1	
10. Affective	0.18	−0.16	−0.01	−0.03	0.03	0.06	0.05	−0.02	−0.08	1
**MAXIMUM LIKELIHOOD ESTIMATED SIGMA BETWEEN CORRELATION MATRIX**										
1. Visual	1									
2. Speech	−0.22	1								
3. Intended	0.38	−0.30	1							
4. Structured	0.52	0.005	0.25	1						
5. Realistic	0.02	0.25	−0.32	0.13	1					
6. Concrete	0.09	0.21	−0.24	−0.08	0.60	1				
7. Important	0.01	0.26	−0.34	0.22	−0.20	−0.14	1			
8. Goals	−0.05	0.23	−0.06	0.12	−0.12	−0.29	0.74	1		
9. Repetitive	0.27	0.16	0.05	0.36	−0.39	−0.22	0.84	0.79	1	
10. Affective	0.35	−0.11	0.27	0.41	0.05	0.02	0.11	0.15	0.14	1
**INTRACLASS CORRELATION**										
	0.22	0.23	0.39	0.26	0.33	0.29	0.23	0.19	0.23	0.33

The next step of MEFA is to partition the total correlation matrix into within and between components. The precise statistical methods to obtain these components are detailed elsewhere (Muthén, 1994; D'Haenens et al., 2010) and the two resulting matrices should be interpreted as follows: if two variables are highly correlated in the between (inter-individual) correlation matrix, this indicates that people who are, on average, high on one variable also tend to be high, on average, on the other variable. On the other hand, if two variables are highly correlated in the within (intra-individual) matrix, this indicates that higher scores (relative to a person's mean) on the first variable tend to co-occur with higher scores (relative to a person's mean) on the second variable within individuals (Reise et al., 2005). The within and between correlation matrices for the present data are shown in Table 1.

**Figure 2:**
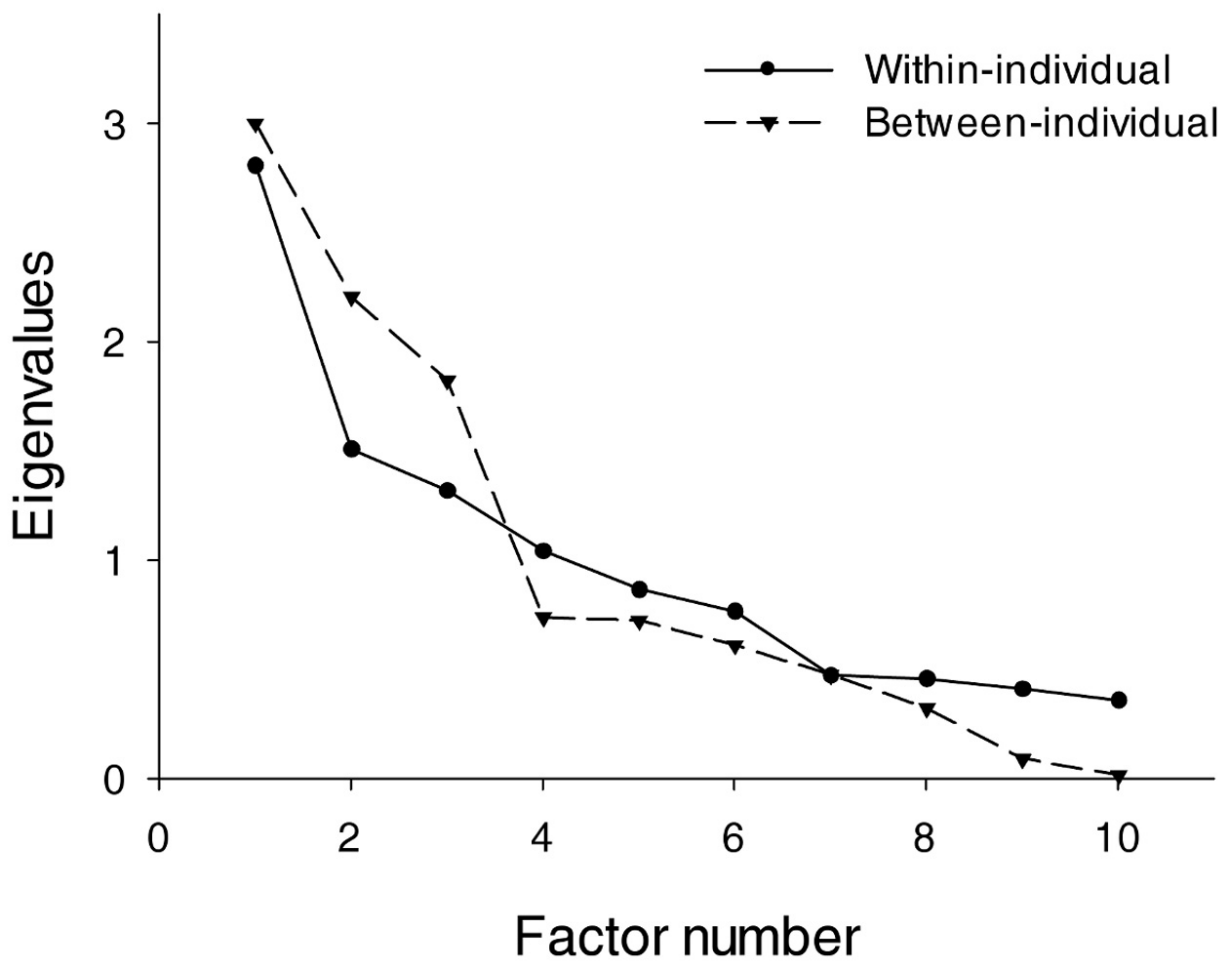
**Scree-plot for the multilevel exploratory factor analysis**. Note: eigenvalues for the MEFA performed at the within- and between-participant level.

Finally, the last step of MEFA is to conduct ordinary factor analyses for each correlation matrix separately. The maximum likelihood EFA performed on the within correlations matrix revealed four factors with eigenvalues above one (respectively, 2.81, 1.51, 1.32, and 1.04; the scree plot is presented in Figure 2), explaining 66.8% of the variance. The resulting four-component solution using an Oblimin (oblique) rotation is presented in Table 2 with the individual component loading for each variable included, as well as the correlations between the factors. We used a rotation that allows the factors to be correlated to avoid the distortions that can occur by forcing an orthogonal rotation onto the data (Reise et al., 2005). The first factor corresponded to the representational format of the thoughts, with dimensions with the strongest loading being, in opposite directions, visual imagery and inner speech. The second factor can be interpreted as reflecting the personal relevance of the thought, with dimensions that loaded most heavily on this factor being the “importance of the thought's content,” “relationship of the thought's content with the participant's personal goals,” and “repetitive occurrence of the thought in the participant's daily life.” The third factor corresponded to the realistic and concrete character of the thought's content. Finally, the dimension that loaded into Factor 4 was the structured aspect of the thought (i.e., whether it was part of a reasoning process or a structured succession of thoughts). Although the affective valence and intentional dimension of the thoughts showed moderate loadings on Factor 1 and Factor 4 respectively, their loadings were weaker than those of other variables. The four factors were only weakly or moderately correlated to each other (see Table 2).

**Table 2:** **Pattern matrix indicating loadings of the TCQ items on the four factors identified at the within (intra-individual) level and correlations between the factors**.

**TCQ items**	**Factor 1**	**Factor 2**	**Factor 3**	**Factor 4**
**ROTATED LOADINGS**				
1. Visual	**−0.747**	0.026	0.001	0.096
2. Speech	**0.724**	0.060	0.025	0.153
3. Intended	0.070	0.006	0.009	0.327
4. Structured	0.178	0.094	−0.017	**0.409**
5. Realistic	0.006	0.102	**0.718**	−0.089
6. Concrete	0.008	−0.080	**0.742**	0.070
7. Important	−0.077	**0.839**	0.048	0.051
8. Goals	0.117	**0.683**	0.047	0.029
9. Repetitive	0.048	**0.724**	−0.093	−0.088
10. Affective	−0.320	0.065	0.057	0.159
**FACTOR CORRELATIONS**				
Factor 1	1			
Factor 2	0.349	1		
Factor 3	0.101	0.254	1	
Factor 4	0.304	0.180	0.204	1

Factor loadings exceeding 0.4 are highlighted in bold.

The maximum likelihood EFA performed on the between correlations matrix revealed three factors with eigenvalues above one (respectively, 3.00, 2.21, and 1.82; the scree plot is presented in Figure 2), explaining 70.3% of the variance. The resulting three-component solution using an Oblimin (oblique) rotation is presented in Table 3 with the individual component loading for each variable included, as well as the correlations between the factors. The dimensions that loaded onto Factor 1 were visual imagery, affective valence, and the intentional and structured aspect of the thoughts. The second factor was similar as for the within-individual analysis and reflected the personal relevance of the thoughts, with dimensions that loaded most strongly being the “importance of the thought's content,” “relationship of the thought's content with the participant's personal goals,” and “repetitive occurrence of the thought in the participant's daily life.” The third factor was also similar as for the within-individual analysis and corresponded to the realistic and concrete character of the thought's content. Inner speech was not specifically related to any of the three factors. The correlations between the three between-factors were negligible.

**Table 3:** **Pattern matrix indicating loadings of the TCQ items on the three factors identified at the between (inter-individual) level and correlations between the factors**.

**TCQ items**	**Factor 1**	**Factor 2**	**Factor 3**
**ROTATED LOADINGS**			
1. Visual	**0.802**	0.068	0.107
2. Speech	−0.251	0.295	0.307
3. Intended	**0.586**	−0.326	−0.398
4. Structured	**0.640**	0.249	0.234
5. Realistic	0.077	−0.121	**0.962**
6. Concrete	0.052	−0.046	**0.613**
7. Important	−0.121	**0.947**	0.055
8. Goals	−0.060	**0.817**	−0.029
9. Repetitive	0.249	**0.926**	−0.192
10. Affective	**0.440**	0.052	0.081
**FACTOR CORRELATIONS**			
Factor 1	1		
Factor 2	0.072	1	
Factor 3	−0.128	−0.133	1

Factor loadings exceeding 0.4 are highlighted in bold.

#### Characterization of future-oriented mind-wandering along the factorial dimensions

**Figure 3:**
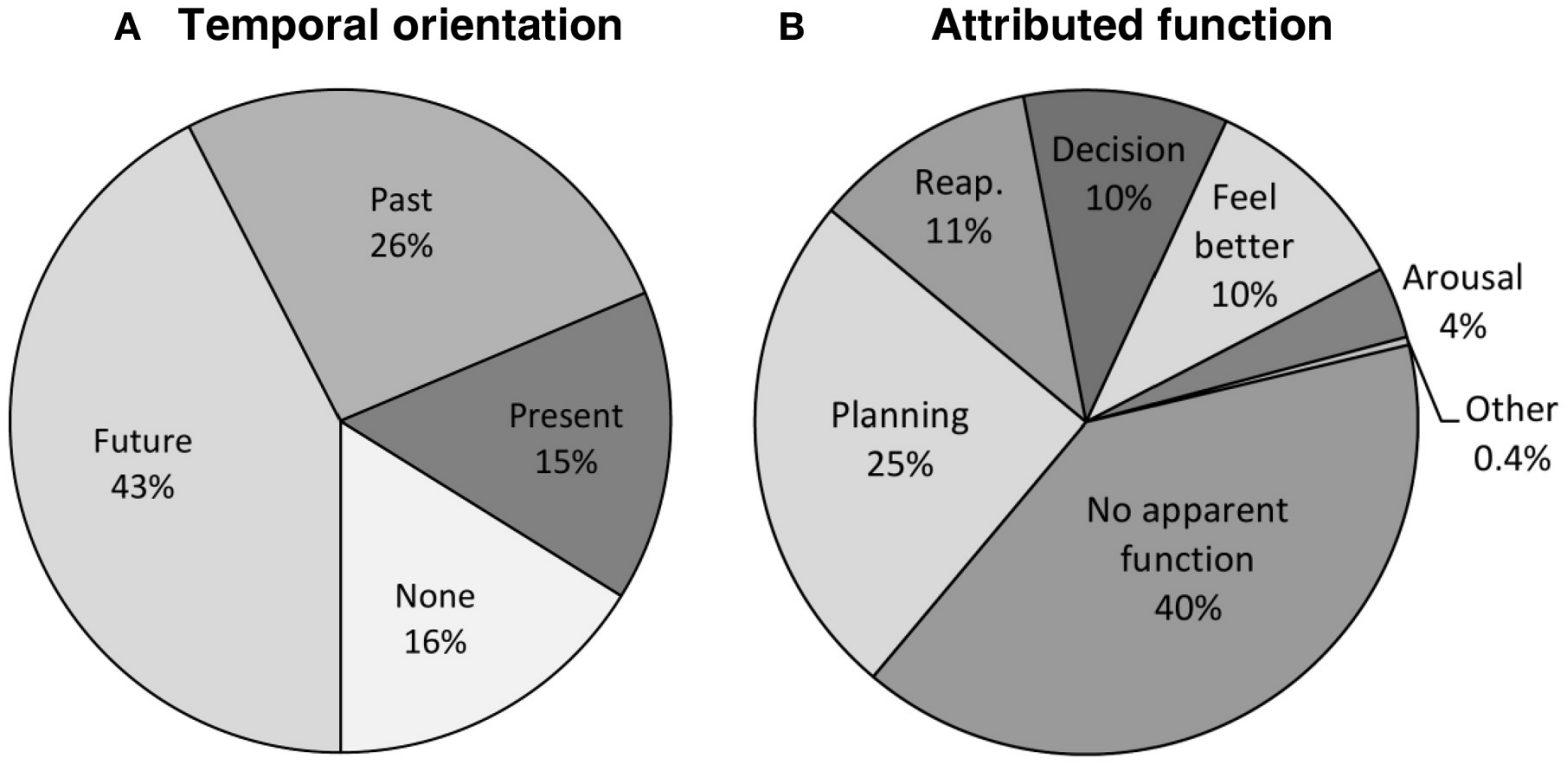
**Distribution of mind-wandering episodes**. Note: Panels **(A)** and **(B)** respectively represent the distribution of the 740 mind-wandering episodes according to their temporal orientation and attributed function; reap. = reappraising a situation; panel **(A)** shows the expected prospective bias of mind-wandering and panel **(B)** reveals that planning was the function most commonly attributed to mind-wandering, although a substantial part of episodes were perceived as not possessing any particular function.

**Figure 4:**
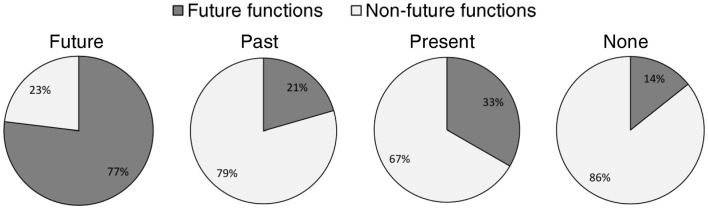
**Distribution of future and non-future functions according to the temporal orientation of mind wandering episodes**. Note: future- and goal-oriented functions were mostly attributed to temporally future-oriented mind-wandering episodes rather than to episodes with other temporal orientation or no precise temporal orientation.

The preceding within-participant factor analysis shows that the phenomenology of individual mind-wandering episodes can be characterized along four key dimensions that correspond to the representational format of the thought, its personal relevance, realistic/concrete character, and structuration. Next, we examined whether these four dimensions vary as a function of the temporal orientation and perceived functions of reported thoughts. The distribution of the mind-wandering episodes according to their temporal orientation is presented in Figure 3A. As we were interested in determining whether and how future-oriented mind-wandering episodes differ from those with other temporal orientations, the 740 mind-wandering episodes were pooled into two categories (future-oriented vs. non-future-oriented) for subsequent analyses^1^. With regard to perceived functions, the distribution of the mind-wandering episodes according to their attributed function is detailed in Figure 3B. Our interest here was in distinguishing between future- and goal-oriented functions (i.e., to make a decision/solve a problem, to plan something, and to reappraise a situation) and functions that are not particularly future-oriented or goal-directed (i.e., to try to feel better, to keep oneself aroused, daydreams with no function or other, non-listed functions) and, therefore, the 740 mind-wandering episodes were pooled into these two categories (i.e., future-oriented functions vs. non-future-oriented functions) for subsequent analyses. The distribution of these two categories of functions according to the temporal orientation of mind-wandering episodes is presented in Figure 4.

For each mind-wandering episode, the dimensions that characterized each of the four factors identified in the within-participant MEFA (i.e., dimensions with coefficients >0.50 in the within correlation matrix and that loaded onto the same factor of the within level of the MEFA) were aggregated into a single score (for the index of representational format, scores on the inner speech items were first reversed, as they correlated negatively with scores on visual imagery). The two dimensions that did not load on any of the four factors (i.e., affective valence and intentional character) were analyzed separately. The proportion of the total variance that was due to within-participant differences (i.e., variation among thoughts) was 81%, 94%, 81%, 88%, 73%, and 91% for representational format, personal relevance, realism/concreteness, structuration, intention, and affect, respectively. To examine whether each phenomenological dimension differed as a function of temporal orientation, we fitted a random intercept multilevel model (using MLwiN; Rasbash et al., 2009) with the index of the phenomenological dimension as dependent variable and temporal orientation (coded as a dummy variable with 0 = future-oriented and 1 = non-future-oriented) as an explanatory variable. The results of these analyses are shown in Table 4. A likelihood ratio (LR) test indicated a significant effect of temporal orientation for representational format, showing that future-oriented thoughts involved less visual imagery/more inner speech than non-future-oriented thoughts^2^. The effect of temporal orientation was also significant for the index of personal relevance, the realistic and concrete character of thoughts, their structured aspect, and intentional dimension: future-oriented thoughts were more self-relevant, more realistic/concrete, more structured, and more intentional than non-future-oriented thoughts. For affective valence, the effect of temporal orientation was not significant.

**Table 4:** **Effects of temporal orientation (future vs. non-future) on the phenomenological dimensions of mind-wandering**.

**Phenomenological dimension**	**Future**		**Non-future**		**Coefficient**	***LR* (1 d.f.)**	***p***
	***M***	***SD***	***M***	***SD***			
Representational format	3.48	1.55	4.88	1.79	1.24 (0.12)	95	<0.001
Personal relevance	4.12	1.51	2.87	1.56	−1.29 (0.12)	113	<0.001
Realism/concreteness	6.07	1.12	5.41	1.57	−0.66 (0.10)	41	<0.001
Structured	3.00	1.78	2.75	1.79	−0.29 (0.13)	4.66	0.03
Intended	2.37	1.49	2.09	1.39	−0.35 (0.10)	12	<0.001
Affect	0.57	1.51	0.46	1.52	−0.15 (0.11)	1.65	0.20

The coefficients represent contrasts with the reference category (the future orientation); standard errors are shown in parenthesis.

Similar multilevel analyses were performed to investigate the influence of perceived functions on each phenomenological dimension of the 740 mind-wandering episodes. The results of these analyses are shown in Table 5. Mind-wandering episodes with future-oriented and goal-directed functions involved less visual imagery/more inner speech than mind-wandering episodes with non-future-oriented functions. The effect of perceived function was also significant for personal relevance, realism/concreteness, structuration, and intention, with each of these dimensions being higher for thoughts with future-oriented functions. Lastly, there was a significant effect of perceived function on affective valence, showing that thoughts with non-future-oriented functions were judged as being more positive than thoughts with future-oriented functions; it should be noted, however, that both kinds of thoughts were, on average, quite neutral (i.e., close to the middle point of the scale).

**Table 5:** **Effects of perceived function (future-oriented vs. non-future-oriented) on the phenomenological dimensions of mind-wandering**.

**Phenomenological dimension**	**Future functions**		**Non-future functions**		**Coefficient**	***LR* (1 d.f.)**	***p***
	***M***	***SD***	***M***	***SD***			
Representational format	3.53	1.66	4.92	1.72	1.33 (0.12)	116	<0.001
Personal relevance	4.18	1.49	2.75	1.50	−1.47 (0.11)	159	<0.001
Realism/concreteness	6.02	1.15	5.41	1.58	−0.68 (0.10)	46	<0.001
Structured	3.13	1.81	2.63	1.75	−0.54 (0.13)	17	<0.001
Intended	2.33	1.44	2.10	1.43	−0.34 (0.10)	12	<0.001
Affect	0.22	1.58	0.74	1.41	0.48 (0.11)	19	<0.001

The coefficients represent contrasts with the reference category (future-oriented functions); standard errors are shown in parenthesis.

**Figure 5:**
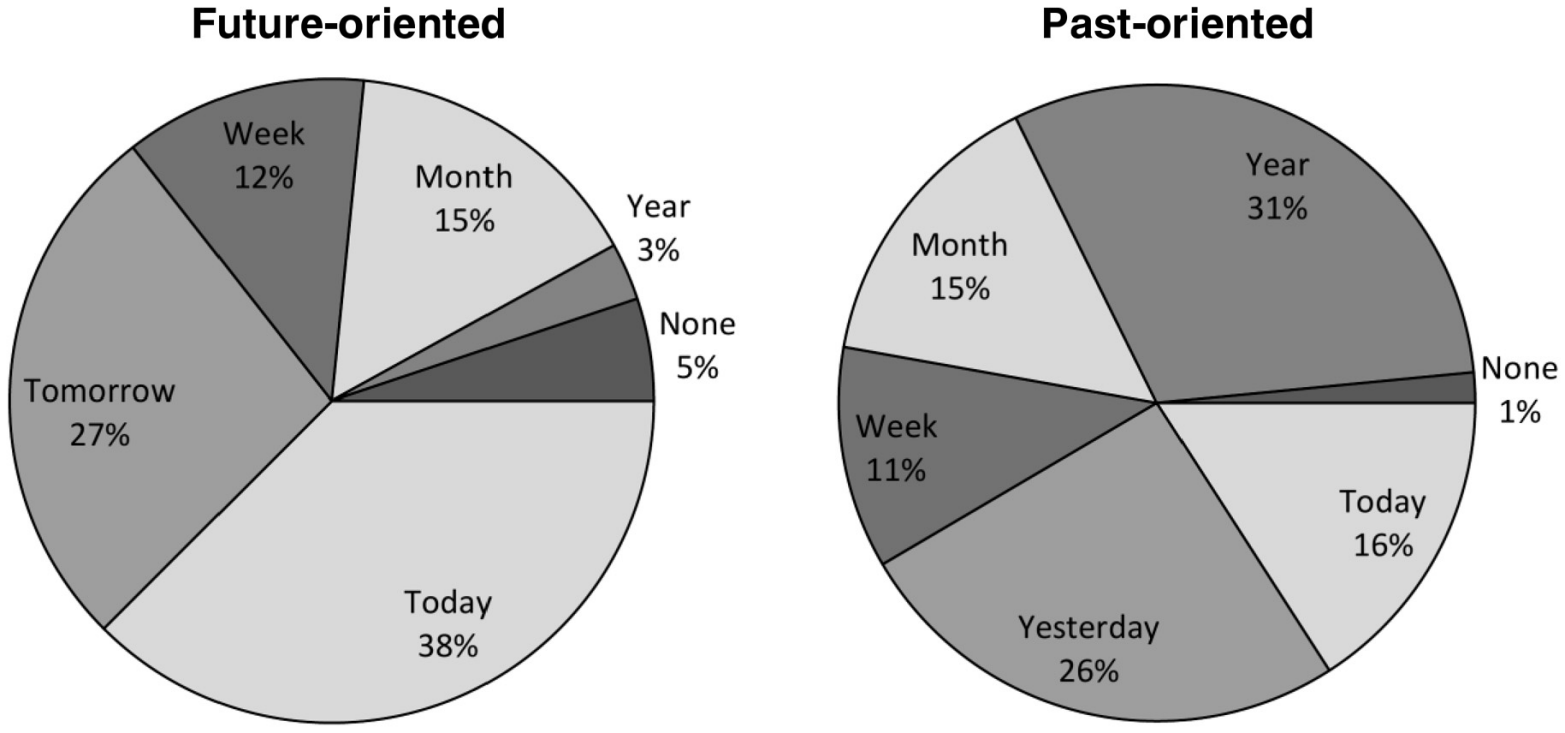
**Temporal distance of mind-wandering episodes according to their temporal orientation**. Note: most future-oriented mind-wandering episodes were related to the close future (later today or in the next 7 days) whereas past-oriented episodes were more equally distributed across the different temporal distances; day = before/later in the present day; week = between yesterday/tomorrow and the past/next 7 days; month = between 1 week and 1 month in the past/future; year = between 1 month and 1 year in the past/future; year + = more than 1 year away in the past/future; none = no precise temporal distance.

Finally, we examined whether the phenomenological dimensions of future-oriented mind-wandering episodes differed as a function of their temporal distance. The distribution of the 312 future- and 195 past-oriented mind-wandering episodes according to their temporal distance is presented in Figure 5. To analyze the effect of temporal distance for the future-oriented episodes, the data were pooled into two categories to have a sufficient number of thoughts per category: close future (combining thoughts referring to the same day and thoughts referring to a time between tomorrow and the next 7 days) and distant future (combining all other temporal distance categories, except the no precise temporal location category which was excluded from the analyses). Multilevel analyses (see Table 6) with each phenomenological dimension as dependent variable and temporal distance (coded as a dummy variable with 0 = close future and 1 = distant future) as predictor variable showed that mind-wandering episodes referring to the distant future were more self-relevant, more structured, and less concrete/realistic than mind-wandering episodes referring to the close future; temporal distance did not significantly influence the representational format, intentional character, and affective valence of thoughts.

**Table 6:** **Effects of temporal distance (close future vs. far future) on the phenomenological dimensions of future-oriented mind-wandering**.

**Phenomenological dimension**	**Close future**		**Far future**		**Coefficient**	***LR* (1 d.f.)**	***p***
	***M***	***SD***	***M***	***SD***			
Representational format	3.43	1.57	3.51	1.54	0.03 (0.19)	0.03	0.86
Personal relevance	3.85	1.41	4.62	1.58	0.77 (0.18)	18	<0.001
Realism/concreteness	6.27	0.94	5.74	1.27	−0.53 (0.12)	19	<0.001
Structured	2.81	1.67	3.33	1.96	0.61 (0.20)	8.90	0.003
Intended	2.43	1.61	2.23	1.22	−0.25 (0.17)	2.24	0.13
Affect	0.57	1.39	0.66	1.67	0.06 (0.18)	0.12	0.73

The coefficients represent contrasts with the reference category (close future); standard errors are shown in parenthesis.

## Discussion

Our aims in this study were to characterize the factorial structure of various phenomenological characteristics of mind-wandering that had been investigated in previous studies (Antrobus et al., 1970; Teasdale et al., 1993; Giambra, 1995; Stuyven and Van Der Goten, 1995; Forster and Lavie, 2009; Marchetti et al., 2012; Song and Wang, 2012) and to determine whether future-oriented mind-wandering episodes differ from other classes of mind-wandering along the identified factorial dimensions.

The MEFA allowed us to determine the factorial structure of mind-wandering episodes at both the within- and between-participant levels. At the within-participant level, the phenomenological features of mind-wandering were structured in four factors. The first factor corresponded to the representational format of the reported thoughts. Inner speech and visual imagery are two fundamental dimensions of inner experience (Klinger and Cox, 1987; Heavey and Hurlburt, 2008; Delamillieure et al., 2010), and the present results showed that these two dimensions were negatively correlated with each other, indicating that mind-wandering episodes usually involved only a single type of representational format. The second factor corresponded to the personal significance of the mind-wandering episode, with high factor loadings for personal importance, relationship with personal goals, and frequency of occurrence in daily life; this finding provides support to the view that personal goals and concerns that manifest repeatedly in daily life are important determinants of mind-wandering (Klinger, 1978; Klinger et al., 1980; Gold and Reilly, 1985). The third factor indicated that mind-wandering episodes differed in their realism and concreteness, and the fourth and final factor corresponded to the structuration of mind-wandering episodes in sequences of thoughts; similar dimensions have been reported to characterize the phenomenology of thought flow in daily life (Klinger and Cox, 1987; Klinger, 2009).

At the between-participant level, the phenomenological features of mind-wandering were structured in three factors, with two of them (personal relevance and realism/concreteness) being identical to those found at the within-participant level. Thus, the dimensions constituting each of these two factors tended to vary together both within participants (e.g., thoughts scored higher on one dimension of personal relevance relative to the person's mean tend to be scored higher on the other dimensions of this factor) and between participants (e.g., individuals with higher mean scores on one dimension of personal relevance tend to possess higher mean scores on the others dimensions of this factor). On the other hand, the third factor of the between analysis was not superimposable to any of the factors identified in the within analysis; it was characterized by high factor loadings for visual imagery, affective valence, and the intentional and structured aspects of mind-wandering episodes. Thus, whereas when taken individually mind-wandering episodes usually involved only a single type of representational format (cf. the results at the within-participants level), we did not find between-participant differences indicating a tendency to experience exclusively visual vs. verbal mind-wandering episodes. Instead, individual differences in the propensity to experience highly visual mind-wandering in the between analysis were associated with the voluntary engagement in structured mind-wandering episodes that are more positively valenced. Although further studies should be conducted to determine the exact nature and implications of this individual difference factor (e.g., by examining its relationships with measures of individual differences such as personality traits, mood states or attentional control abilities), this finding nonetheless demonstrates the importance of distinguishing between within- and between-subject levels for characterizing the phenomenological structure of mind-wandering. This may have important implications for studies that attempt to determine the neural substrates of subjective experience during mind-wandering and the so-called “resting state” (Buckner et al., 2008; Christoff et al., 2009; Stawarczyk et al., 2011b). Indeed, there has been a growing interest in using functional neuroimaging techniques to link subjective experience to patterns of brain activity (i.e., a neurophenomenological approach; Lutz and Thompson, 2003; Rudrauf et al., 2003) but, to date, most studies have investigated variations between participants (e.g., Andrews-Hanna et al., 2010; Doucet et al., 2012). Our findings suggest that linking brain activity to phenomenological features at the within-participant level might provide important additional information.

Having identified the factorial structure of the phenomenological dimensions of mind-wandering at the within-participant level, we then investigated whether prospective mind-wandering episodes differ from other classes of mind-wandering episodes along the identified factors. We found that mind-wandering episodes that were temporally and functionally oriented toward the future involved more inner speech and less visual imagery than non-future-oriented mind-wandering episodes, and were more self-relevant, realistic/concrete, intentional, and part of structured sequences of thoughts. These findings provide novel evidence that prospective mind-wandering possesses a unique phenomenological signature. In terms of representational format, the lower involvement of visual imagery in future-oriented mind-wandering is in line with previous studies on directed future thinking, which have shown that future thoughts generally contain fewer sensory details (including visual details) than past thoughts (D'Argembeau and Van Der Linden, 2004, 2006; Berntsen and Bohn, 2010). On the other hand, inner speech seems a privileged modality for the expression of prospective mind-wandering. Evidence from thought-sampling studies suggests that inner speech plays an important role in planning, problem solving, and self-regulation (D'Argembeau et al., 2011; Morin et al., 2011). For instance, Morin et al. (2011) found that planning was by far the most frequent self-reported function of inner speech, and D'Argembeau et al. (2011) found that future-oriented thoughts involved in planning and decision making were frequently in the form of inner speech. The current finding that mind-wandering episodes with future-oriented functions (i.e., to make a decision/solve a problem, to plan something, and to reappraise a situation) involved inner speech to a greater extent than non-future-oriented thoughts provides additional evidence for the central role of inner speech in autobiographical planning. Of course, this does not exclude the fact that visual imagery also plays an important role, allowing the construction of detailed simulations of what it would be like to be in specific future events (D'Argembeau and Van Der Linden, 2006). Identifying the precise determinants of the use of different modalities for representing possible futures is an important avenue for future research.

Our finding that prospective mind-wandering episodes were more self-relevant than non-prospective forms of mind-wandering is also in line with studies on directed future thinking (D'Argembeau and Van Der Linden, 2006; Addis et al., 2008; Berntsen and Bohn, 2010) and provides support for the view that an important function of mind-wandering is to enable the anticipation and planning of personally relevant future goals (Baird et al., 2011; Stawarczyk et al., 2011a). On the other hand, the finding that future-oriented mind-wandering episodes were perceived as more realistic and concrete than other kinds of episodes may, at first sight, seem contradictory to previous research on future thinking. Several studies have indeed found that mental representations of future events are usually more vague and are guided by general life scripts to a larger extent than memories of past events (D'Argembeau and Van Der Linden, 2004, 2006; Berntsen and Bohn, 2010). It is important to note, however, that the future- and past-oriented mind-wandering episodes that were collected in the current study were not matched to each other in terms of temporal distance: only 3% of future-oriented mind-wandering episodes involved a time period more than 1 year away, compared to 31% for past-oriented mind-wandering. Therefore, given that past and future events are represented more abstractly with increasing temporal distance (D'Argembeau and Van Der Linden, 2004; Addis et al., 2008; Liberman and Trope, 2008; D'Argembeau et al., 2011), it could simply be that the higher realism/concreteness of future-oriented mind-wandering episodes resulted from the effect of temporal distance; indeed, in the present study, we found that mind-wandering episodes referring to the distant future were less concrete/realistic than mind-wandering episodes referring to the close future. Be that as it may, the subjective experience of realism associated with imagined prospects may play an important role in determining the impact of mind-wandering episodes on one's decisions and behavior (Johnson and Sherman, 1990; Roese and Sherman, 2007).

Interestingly, we also found that future-oriented mind-wandering episodes were more structured and intended than the other classes of mind-wandering. This might be related to the fact that prospective mind-wandering frequently involves autobiographical planning (Baird et al., 2011). Thinking about ways of approaching and realizing personal goals often involves simulating a sequence of successive interdependent events and actions that are causally linked to each other. The more structured and intended nature of future-oriented mind-wandering episodes might reflect such autobiographical planning processes. These dimensions might also in part explain why prospective mind-wandering makes more demands on executive resources than non-future-oriented mind-wandering (Smallwood et al., 2009; Baird et al., 2011). Executive control processes may indeed play an important role in relating and coordinating envisioned possibilities in order to construct structured trains of thoughts that can aid finding the best route for attaining personal goals (Spreng et al., 2010; Gerlach et al., 2011).

To conclude, the present findings provide new insights as to what differentiate prospective from non-prospective forms of mind-wandering. In particular, our results show that future-oriented mind-wandering episodes preferentially involve inner speech and are more self-relevant, concrete/realistic, structured and intended than non-future-oriented mind-wandering episodes. Taken together, these features might in part explain why some types of mind-wandering may provide more benefits than others (Mooneyham and Schooler, 2013). An important function of prospective mind-wandering might be to manage personal goals and plan effective ways of attaining desired prospects. The construction of structured sequences of goal-directed thoughts, perhaps mainly via the use of inner speech, is likely an essential ingredient of this process.

### Conflict of interest statement

The authors declare that the research was conducted in the absence of any commercial or financial relationships that could be construed as a potential conflict of interest.

## Footnotes

^1^Although our main interest in this study was to compare future-oriented with non-future-oriented mind wandering episodes, it would have also been interesting to investigate whether the three types of non-future-oriented episodes (i.e., episodes referring to the past, the present, or with no precise temporal orientation) differ from each other. However, only 25 out of the 67 participants in this study reported at least one episode for each of these categories. Given this loss of statistical power, we decided to only present the analyses on the pooled data for the non-future mind-wandering categories. We nonetheless performed additional analyses that specifically contrasted future- vs. past-oriented mind-wandering episodes. These analyses were conducted on 454 mind-wandering episodes nested within the 46 participants who reported at least one future-oriented and one past-oriented mind-wandering episode. The results of these analyses were for the most part the same as those obtained when comparing future-oriented mind-wandering with the pooled non-future category. The only difference was that future-oriented episodes were not significantly more structured than past-oriented episodes (coefficient = −0.17; *SE* = 0.17; *p* = 0.32; Future = 3.04 ± 1.73, Past = 2.99 ± 1.86).

^2^For completeness, we also examined each of these two dimensions individually. These analyses showed that future-oriented mind-wandering episodes involved both more inner speech [Coeff. = −1.40 (0.14); *LR* = 94.51; *p* < 0.001; Future = 4.58 ± 1.89; Non-future = 3.04 ± 2] and less visual imagery [Coeff. = 1.06 (0.15); *LR* = 50.33; *p* < 0.001; Future = 3.55 ± 1.91, Non-future = 4.80 ± 2.17] than non-future-oriented episodes.

## References

[B1] Addis D. R. Wong A. T. Schacter D. L. (2008). Age-related changes in the episodic simulation of future events. Psychol. Sci. 19, 33–41 10.1111/j.1467-9280.2008.02043.x 18181789

[B2] Andrews-Hanna J. R. (2012). The brain's default network and its adaptive role in internal mentation. Neuroscientist 18, 251–270 10.1177/1073858411403316 21677128 PMC3553600

[B3] Andrews-Hanna J. R. Reidler J. S. Huang C. Buckner R. L. (2010). Evidence for the default network's role in spontaneous cognition. J. Neurophysiol. 104, 322–335 10.1152/jn.00830.2009 20463201 PMC2904225

[B4] Antrobus J. S. Singer J. L. Goldstein S. Fortgang M. (1970). Mindwandering and cognitive structure. Trans. N.Y. Acad. Sci. 32, 242–252 10.1111/j.2164-0947.1970.tb02056.x 5265228

[B5] Antrobus J. S. Singer J. L. Greenberg S. (1966). Studies in the stream of consciousness: experimental enhancement and suppression of spontaneous cognitive processes. Percept. Motor Skills 23, 399–417 10.2466/pms.1966.23.2.399

[B6] Baird B. Smallwood J. Schooler J. W. (2011). Back to the future: autobiographical planning and the functionality of mind-wandering. Conscious. Cogn. 20, 1604–1611 10.1016/j.concog.2011.08.007 21917482

[B7] Barron E. Riby L. M. Greer J. Smallwood J. (2011). Absorbed in thought: the effect of mind wandering on the processing of relevant and irrelevant events. Psychol. Sci. 22, 596–601 10.1177/0956797611404083 21460338

[B8] Berntsen D. Bohn A. (2010). Remembering and forecasting: the relation between autobiographical memory and episodic future thinking. Mem. Cogn. 38, 265–278 10.3758/MC.38.3.265 20234017

[B9] Braboszcz C. Delorme A. (2011). Lost in thoughts: neural markers of low alertness during mind wandering. Neuroimage 54, 3040–3047 10.1016/j.neuroimage.2010.10.008 20946963

[B10] Buckner R. L. Andrews-Hanna J. R. Schacter D. L. (2008). “The brain's default network: anatomy, function, and relevance to disease,” in The Year in Cognitive Neuroscience 2008, eds Kingstone A. Miller M. B. (Malden, MA: Blackwell Publishing), 1–38 10.1196/annals.1440.011 18400922

[B11] Christoff K. (2012). Undirected thought: neural determinants and correlates. Brain Res. 1428, 51–59 10.1016/j.brainres.2011.09.060 22071565

[B12] Christoff K. Gordon A. Smith R. (2011). “The role of spontaneous thought in human cognition,” in Neuroscience of Decision Making, eds Vartanian O. Mandel D. R. (Hove, NY: Psychology Press), 259–284

[B13] Christoff K. Gordon A. M. Smallwood J. Schooler J. W. Smith R. (2009). Experience sampling during fMRI reveals default network and executive system contributions to mind wandering. Proc. Natl. Acad. Sci. U.S.A. 106, 8719–8724 10.1073/pnas.0900234106 19433790 PMC2689035

[B14] D'Argembeau A. Renaud O. Van Der Linden M. (2011). Frequency, characteristics, and functions of future-oriented thoughts in daily life. Appl. Cogn. Psychol. 25, 96–103 10.1002/acp.1647

[B15] D'Argembeau A. Van Der Linden M. (2004). Phenomenal characteristics associated with projecting oneself back into the past and forward into the future: influence of valence and temporal distance. Conscious. Cogn. 13, 844–858 10.1016/j.concog.2004.07.007 15522635

[B16] D'Argembeau A. Van Der Linden M. (2006). Individual differences in the phenomenology of mental time travel: the effect of vivid visual imagery and emotion regulation strategies. Conscious. Cogn. 15, 342–350 10.1016/j.concog.2005.09.001 16230028

[B18] Delamillieure P. Doucet G. Mazoyer B. Turbelin M. R. Delcroix N. Mellet E. (2010). The resting state questionnaire: an introspective questionnaire for evaluation of inner experience during the conscious resting state. Brain Res. Bull. 81, 565–573 10.1016/j.brainresbull.2009.11.014 20003916

[B17] D'Haenens E. Van Damme J. Onghena P. (2010). Multilevel exploratory factor analysis: illustrating its surplus value in educational effectiveness research. School Effect. School Improve. 21, 209–235 10.1080/09243450903581218

[B19] Doucet G. Naveau M. Petit L. Zago L. Crivello F. Jobard G. (2012). Patterns of hemodynamic low-frequency oscillations in the brain are modulated by the nature of free thought during rest. Neuroimage 59, 3194–31200 10.1016/j.neuroimage.2011.11.059 22155378

[B20] Fell J. (2012). I think, therefore I am (unhappy). Front. Hum. Neurosci. 6:132 10.3389/fnhum.2012.00132 22623916 PMC3353261

[B21] Forster S. Lavie N. (2009). Harnessing the wandering mind: the role of perceptual load. Cognition 111, 345–355 10.1016/j.cognition.2009.02.006 19327760 PMC2706319

[B22] Gerlach K. D. Spreng R. N. Gilmore A. W. Schacter D. L. (2011). Solving future problems: default network and executive activity associated with goal-directed mental simulations. Neuroimage 55, 1816–1824 10.1016/j.neuroimage.2011.01.030 21256228 PMC3855008

[B23] Giambra L. M. (1995). A laboratory method for investigating influences on switching attention to task-unrelated imagery and thought. Conscious. Cogn. 4, 1–21 10.1006/ccog.1995.1001 7497092

[B24] Gold S. R. Reilly J. P. (1985). Daydreaming, current concerns and personality. Imag. Cogn. Pers. 5, 117–125 10.2190/BR6K-0VUW-44GC-VLA4

[B25] Heavey C. L. Hurlburt R. T. (2008). The phenomena of inner experience. Conscious. Cogn. Int. J. 17, 798–810 10.1016/j.concog.2007.12.006 18258456

[B26] Johnson M. K. (1988). Reality monitoring: an experimental phenomenological approach. J. Exp. Psychol. Gen. 117, 390–394 10.1037/0096-3445.117.4.390

[B27] Johnson M. K. Foley M. A. Suengas A. G. Raye C. L. (1988). Phenomenal characteristics of memories for perceived and imagined autobiographical events. J. Exp. Psychol. Gen. 117, 371–376 10.1037/0096-3445.117.4.371 2974863

[B28] Johnson M. K. Sherman S. J. (1990). “Constructing and reconstructing the past and the future in the present,” in Handbook of Motivation and Cognition: Foundations of Social Behavior, Vol. 2, eds Higgins E. T. Sorrentino R. M. (New York, NY: Guilford Press), 482–526

[B29] Kam J. W. Y. Dao E. Farley J. Fitzpatrick K. Smallwood J. Schooler J. W. (2011). Slow fluctuations in attentional control of sensory cortex. J. Cogn. Neurosci. 23, 460–470 10.1162/jocn.2010.21443 20146593

[B30] Kane M. J. Brown L. H. McVay J. C. Silvia P. J. Myin-Germeys I. Kwapil T. R. (2007). For whom the mind wanders, and when: an experience-sampling study of working memory and executive control in daily life. Psychol. Sci. 18, 614–621 10.1111/j.1467-9280.2007.01948.x 17614870

[B31] Kane M. J. McVay J. C. (2012). What mind wandering reveals about executive-control abilities and failures. Curr. Dir. Psychol. Sci. 21, 348–354 10.1177/0963721412454875

[B32] Killingsworth M. A. Gilbert D. T. (2010). A wandering mind is an unhappy mind. Science 330:932 10.1126/science.1192439 21071660

[B33] Klinger E. (1978). “Modes of normal conscious flow,” in The Stream of Consciousness, eds Pope K. S. Singer J. L. (New York, NY: Plenum), 225–258

[B34] Klinger E. (1999). “Thought flow: properties and mechanisms underlying shifts in content,” in At Play in the Fields of Consciousness, eds Singer J. A. Salovey P. (Mahwah, NJ: Lawrence Erlbaum Associates Publishers), 29–50

[B35] Klinger E. (2009). “Daydreaming and fantasizing: thought flow and motivation,” in Handbook of Imagination and Mental Simulation, eds Markman K. D. Klein W. M. P. Suhr J. A. (New York, NY: Psychology Press), 225–239

[B36] Klinger E. Barta S. G. Maxeiner M. E. (1980). Motivational correlates of thought content frequency and commitment. J. Person. Soc. Psychol. 39, 1222–1237 10.1037/h0077724

[B37] Klinger E. Cox W. M. (1987). Dimensions of thought flow in everyday life. Imag. Cogn. Pers. 7, 105–128 10.2190/7K24-G343-MTQW-115V

[B38] Liberman N. Trope Y. (2008). The psychology of transcending the here and now. Science 322, 1201–1205 10.1126/science.1161958 19023074 PMC2643344

[B39] Lutz A. Thompson E. (2003). Neurophenomenology: integrating subjective experience and brain dynamics in the neuroscience of consciousness. J. Conscious. Stud. 10, 31–52

[B40] Macleod A. K. Byrne A. (1996). Anxiety, depression, and the anticipation of future positive and negative experiences. J. Abnorm. Psychol. 105, 286–289 10.1037/0021-843X.105.2.286 8723011

[B41] Marchetti I. Koster E. H. De Raedt R. (2012). Mindwandering heightens the accessibility of negative relative to positive thought. Conscious. Cogn. 21, 1517–1525 10.1016/j.concog.2012.05.013 22726693

[B42] McVay J. C. Kane M. J. (2009). Conducting the train of thought: working memory capacity, goal neglect, and mind wandering in an executive-control task. J. Exp. Psychol. Learn. Mem. Cogn. 35, 196–204 10.1037/a0014104 19210090 PMC2750806

[B43] McVay J. C. Kane M. J. (2010). “Adrift in the stream of thought: the effects of mind wandering on executive control and working memory capacity,” in Handbook of Individual Differences in Cognition: Attention, Memory, and Executive Control, eds Gruszka A. Matthews G. Szymura B. (New York, NY: Springer), 321–334

[B44] McVay J. C. Kane M. J. (2012). Drifting from slow to “d'oh!”: working memory capacity and mind wandering predict extreme reaction times and executive control errors. J. Exp. Psychol. Learn. Mem. Cogn. 38, 525–549 10.1037/a0025896 22004270 PMC3395723

[B45] Mooneyham B. W. Schooler J. W. (2013). The costs and benefits of mind-wandering: a review. Can. J. Exp. Psychol. 67, 11–18 10.1037/a0031569 23458547

[B46] Morin A. Uttl B. Hamper B. (2011). Self-reported frequency, content, and functions of inner speech. Proc. Soc. Behav. Sci. 30, 1714–1718 10.1016/j.sbspro.2011.10.331

[B47] Muthén B. O. (1994). Multilevel covariance structure analysis. Soc. Methods Res. 22, 376–398 10.1177/0049124194022003006

[B48] Muthén L. K. Muthén B. O. (1998-2010). Mplus User's Guide, 6th Edn. Los Angeles, CA: Muthén & Muthén

[B49] Rasbash J. Charlton C. Browne W. J. Healy M. Cameron B. (2009). MLwiN Version 2.1. Centre for Multilevel Modelling, University of Bristol.

[B50] Rasmussen A. S. Berntsen D. (2013). The reality of the past versus the ideality of the future: emotional valence and functional differences between past and future mental time travel. Mem. Cogn. 41, 187–200 10.3758/s13421-012-0260-y 23055119

[B51] Reise S. P. Ventura J. Nuechterlein K. H. Kim K. H. (2005). An illustration of multilevel factor analysis. J. Pers. Assess. 84, 126–136 10.1207/s15327752jpa8402_02 15799887

[B52] Robertson I. H. Manly T. Andrade J. Baddeley B. T. Yiend J. (1997). ‘Oops!’: performance correlates of everyday attentional failures in traumatic brain injured and normal subjects. Neuropsychologia 35, 747–758 10.1016/S0028-3932(97)00015-8 9204482

[B53] Roese N. J. Sherman J. W. (2007). “Expectancies,” in Social Psychology: Handbook of Basic Principles, 2nd Edn., eds Higgins E. T. Kruglanski A. W. (New York, NY: Guilford Press), 91–115

[B54] Rudrauf D. Lutz A. Cosmelli D. Lachaux J. P. Le Van Quyen M. (2003). From autopoiesis to neurophenomenology: Francisco Varela's exploration of the biophysics of being. Biol. Res. 36, 27–65 10.4067/S0716-97602003000100005 12795206

[B55] Schooler J. W. Smallwood J. Christoff K. Handy T. C. Reichle E. D. Sayette M. A. (2011). Meta-awareness, perceptual decoupling and the wandering mind. Trends Cogn. Sci. 15, 319–326 21684189 10.1016/j.tics.2011.05.006

[B56] Singer J. L. (1993). Experimental studies of ongoing conscious experience. Ciba Found. Symp. 174, 100–122 8319504

[B57] Smallwood J. (2010). Why the global availability of mind wandering necessitates resource competition: reply to McVay Kane (2010). Psychol. Bull. 136, 202–207 10.1037/a0018673

[B58] Smallwood J. (2011). Mind-wandering while reading: attentional decoupling, mindless reading and the cascade model of inattention. Lang. Linguist. Comp. 5, 63–77 10.1111/j.1749-818X.2010.00263.x

[B59] Smallwood J. (2013). Distinguishing how from why the mind wanders: a process-occurrence framework for self-generated mental activity. Psychol. Bull. 139, 519–535 10.1037/a0030010 23607430

[B60] Smallwood J. Brown K. Baird B. Schooler J. W. (2012). Cooperation between the default mode network and the frontal-parietal network in the production of an internal train of thought. Brain Res. 1428, 60–70 10.1016/j.brainres.2011.03.072 21466793

[B61] Smallwood J. Fishman D. J. Schooler J. W. (2007). Counting the cost of an absent mind: mind wandering as an underrecognized influence on educational performance. Psychon. Bull. Rev. 14, 230–236 10.3758/BF03194057 17694906

[B62] Smallwood J. Nind L. O'Connor R. C. (2009). When is your head at? An exploration of the factors associated with the temporal focus of the wandering mind. Conscious. Cogn. 18, 118–125 10.1016/j.concog.2008.11.004 19121953

[B63] Smallwood J. Schooler J. W. (2006). The restless mind. Psychol. Bull. 132, 946–958 10.1037/0033-2909.132.6.946 17073528

[B64] Smallwood J. Schooler J. W. Turk D. J. Cunningham S. J. Burns P. Macrae C. N. (2011). Self-reflection and the temporal focus of the wandering mind. Conscious. Cogn. 20, 1120–1126 10.1016/j.concog.2010.12.017 21277803

[B65] Song X. Wang X. (2012). Mind wandering in Chinese daily lives - an experience sampling study. PLoS ONE 7:e44423 10.1371/journal.pone.0044423 22957071 PMC3434139

[B66] Spreng R. N. Stevens W. D. Chamberlain J. P. Gilmore A. W. Schacter D. L. (2010). Default network activity, coupled with the frontoparietal control network, supports goal-directed cognition. Neuroimage 53, 303–317 10.1016/j.neuroimage.2010.06.016 20600998 PMC2914129

[B67] Stawarczyk D. Majerus S. Maj M. Van Der Linden M. D'Argembeau A. (2011a). Mind-wandering: phenomenology and function as assessed with a novel experience sampling method. Acta Psychol. 136, 370–381 10.1016/j.actpsy.2011.01.002 21349473

[B68] Stawarczyk D. Majerus S. Maquet P. D'Argembeau A. (2011b). Neural correlates of ongoing conscious experience: both task-unrelatedness and stimulus-independence are related to default network activity. PLoS ONE 6:e16997 10.1371/journal.pone.0016997 21347270 PMC3038939

[B69] Stuyven E. Van Der Goten K. (1995). Stimulus independent thoughts and working memory: the role of the central executive. Psychol. Belgica 35, 241–251

[B70] Teasdale J. D. Proctor L. Lloyd C. A. Baddeley A. D. (1993). Working memory and stimulus-independent thought: effects of memory load and presentation rate. Eur. J. Cogn. Psychol. 5, 417–433 10.1080/09541449308520128

[B71] Watkins E. R. (2008). Constructive and unconstructive repetitive thought. Psychol. Bull. 134, 163–206 10.1037/0033-2909.134.2.163 18298268 PMC2672052

[B72] Watkins E. R. (2010). Level of construal, mind wandering, repetitive thought: reply to McVay and Kane (2010). Psychol. Bull. 136, 198–201 10.1037/a0018563

